# Prevalence, risk factors, and severity of erectile dysfunction following renal transplantation

**DOI:** 10.1007/s11255-024-04299-9

**Published:** 2024-12-02

**Authors:** Adelina Miron, Ionuț Nistor, Corneliu Moroșanu, Lucian Sirițeanu, Catalin Pricop, Dragos Puia, Adrian Covic

**Affiliations:** 1 “Grigore T. Popa” University of Medicine and Pharmacy”, Iasi, Romania; 2 Department of Urology and Renal Transplantation, “Dr. C.I. Parhon” Hospital, Iasi, Romania; 3 Department of Nephrology “Dr. C.I. Parhon” Hospital, Iasi, Romania; 4 Innovation and Technology Transfer Center MAVIS, “Grigore T. Popa” University of Medicine and Pharmacy, Iasi, Romania

**Keywords:** Kidney transplant, Sexual dysfunction, Erectile function, Chronic kidney disease

## Abstract

**Background:**

Sexual dysfunction is common among dialysis and transplant patients. Our study evaluated the prevalence, risk factors, and severity of erectile dysfunction (ED) post-transplant in a single center.

**Methods:**

We conducted a single-centre, observational, non-interventional study of adult male renal graft recipients. Sociodemographic and clinical data were collected, and erectile function was assessed with the International Index of Erectile Function (IIEF) questionnaire.

**Results:**

179 patients transplanted between 1995 and 2021 were enrolled (170 answered the questionnaire). Mild, moderate, and severe ED was noted in 33.5%, 20.6% and 10.6% of cases, respectively. ED prevalence increased with age (42.6% of patients < 40, 47.4% of patients aged 40–60, 78.9% of patients > 60). The total mean IIEF score was 16.32 ± 6.93 (erectile function 19.22 ± 7.9, orgasmic function 6.8 ± 2.9, sexual desire 6.43 ± 2.1, intercourse satisfaction 8.96 ± 3.7, overall satisfaction 6.78 ± 2.6). Age, alcohol consumption, type, time on dialysis pre-transplant, and donor type were significantly associated with erectile dysfunction (p < 0.05). Most patients (93.5%) were treated for comorbidities in addition to immunosuppression. Severe ED was significantly more common among patients taking alpha blockers and non-steroidal anti-inflammatory drugs.

**Conclusions:**

Self-reported erectile dysfunction post renal transplantation seems influenced by age, alcohol intake, dialysis history, donor type and certain drugs, but not by comorbidities (hypertension, diabetes, heart disease).

## Introduction

Kidney chronic disease has a major impact on the body’s overall homeostasis. Dialysis for patients with renal failure decreases both the overall survival rate and the quality of life socially, psychologically, and physically compering with people without kidney disease [1–3].

Erectile dysfunction is more common in dialysis patients than in the general population, for which prevalence data differ across different studies and geographical areas: 52% in the USA [3], to 18.9% in Spain [4], 20% in men older than 50 in France [5], 52% in Swedish patients [6], 14.7% in Brazilian men [7], 19.8% in Australian patients [8], 48.1% in men over 40 years old in Portugal [9]. Based on such findings, erectile dysfunction is considered one of the most common conditions in male patients after the age of 40, and it increased with age [10].

The possible causes of erectile dysfunction in dialysis patients are numerous (vascular, neurological, hormonal, anatomical, pharmacological, psychogenic, traumatic). Apart from age, other risk factors relate to the underlying chronic kidney disease itself, as well as to these patients’, frequently associated comorbidities, such as hypertension, cardiovascular conditions, metabolic syndrome, endocrinological diseases, diabetes, neuropsychological and psychological disorders, unhealthy lifestyle, previous pelvic surgeries, urological conditions, administered medication (antihypertensives, immunosuppressives, diuretics, beta-blockers, etc.) [11, 12].

Renal transplantation greatly improves the quality of life of these patients and, as such, it can also positively influence sexual function. So far, research into patients’ sex lives post-transplant has produced controversial results. While some authors found improved erectile function 6 months or more after renal transplantation, others reported no significant benefits in terms of sexual activity [13–23]. Moreover, studies document “de novo” incidence of erectile dysfunction [24–27].

Chronic kidney diseases (CKD) and ED share common vascular, neurological, hormonal, and psychological risk factors. Increasing evidence suggests that renal failure is associated with anxiety, depression, poor quality of life, all of which can progress to erectile dysfunction [28]. Other than renal failure, diabetes (50%), atherosclerotic diseases (40%), and liver failure (25%) have also been associated with ED in various degrees [29].

At the same time, kidney transplant recipients are considered a subgroup of chronic patients who may enjoy improved health-related quality of life at a level com-parable to the general population, as well as increased long-term survival rates [30]. However, erectile dysfunction sems to persist or even to occur anew in some cases. The literature is insufficient with regard to sexual function in transplanted patients, while risk factors are mounting. For instance, erectile function is increasingly affected by medication e.g., hypogonadism as a side effect of steroids used for immunosuppression, interference with penis vascularity, etc. [31]. Although erectile function is not the most significant indicator of sexual fulfilment for all men receiving renal transplants, it does cause substantial mental stress to many, which further impact familial and social interactions [32].

There is still much to learn about the sexual function of kidney transplant recipients and dialyzed patients, as well as about the impact of renal transplantation on sexuality. Now that patients are able to enjoy better graft survival, longer overall survival, and improved quality of life, erectile (dys)function is becoming a topic of increased interest. Although it is considered a benign disorder, ED has many physical, psychological, and social implications for the patient and their partners [33]. It is important to acknowledge this and, in practice, to identify trans-planted patients with erectile dysfunction, to explore possible causes, and to recom-mend effective treatment accordingly. Positive results can further increase the quality of life for these patients and produce long-term benefits.

### Aims

This study was conducted to assess the prevalence of erectile dysfunction among recipients of renal grafts a year or more after transplantation, seeking to appraise the patients’ own perspectives and to identify the risk factors contributing to erectile dysfunction in these patients. More specifically, the study aimed to identify sexual impairment in adult male renal recipients, to evaluate the profile of transplant recipients with and without erectile dysfunction, and to estimate the prevalence, risk factors, and severity of sexual dysfunction in this category of patients.

## Materials and methods

We conducted a single-center, retrospective, observational study of adult male patients with chronic kidney disease who had received renal grafts during 1995–2021 and were being monitored at the renal transplant unit of “Dr. C.I. Parhon” Hospital from Iași, one of three such transplant centers in Romania covers around 4.5 million people. The data came from patients operated on at this and other centers at least 6 months prior to enrolment. The study was approved by the Ethics Board of the hospital, and all the patients consented in writing.

### Participants and data collection

Apart from providing consent and being of adult age, transplant patients had to have functioning renal allografts at their 6-month and 12-month follow up consultations to be eligible for inclusion in the study. Patients with immediate or late postoperative severe complications (acute or chronic graft rejection), patients whose graft failure required them to return to dialysis, and men with genital malformations incompatible with sexual activity were excluded.

Data collected from the patients’ hospital files included:

•General characteristics: age, biometrics (weight, height), social background (level of education, marital status, employment status).
•Pretransplant clinical data regarding the primary kidney disease, type of impairment, history of dialysis, presence of comorbidities (diabetes, hypertension, viral hepatitis, vascular insufficiency, neurological conditions, urological disease).
•Data regarding the surgery and early post-transplantation period (time since transplant, type of donor, type of vascular anastomosis, surgical complications, type of induction and maintenance immunosuppression).
•Blood and urine laboratory test indicative of renal function, liver function, presence of metabolic syndrome, inflammatory syndrome, proteinuria, etc. at the first evaluation post-discharge (which we considered as baseline data), then again at 6 months and at 12 months after the renal transplantation, as well as on enrolment in 2021.

The patients were enrolled as they presented for their follow up assessments. This is also when they were asked to fill out a standard validated questionnaire for sexual function, namely the International Index for Erectile Function (IIEF) as described by Rosen et al. [34]. The questionnaire was completed at the start of the study in 2021 and again after a 2-year interval in 2023 by the same group of patients. The IIEF domains (erectile function, orgasmic function, sexual desire, intercourse satisfaction, and overall satisfaction) were scored for each responder [34]. Severity scores were calculated to establish the diagnosis of erectile dysfunction at this point (6–12 months after surgery) and the other available patient data were used comprehensively to assess risk. The presence and severity of ED were defined according to the classification system proposed by Rosen et al. and Cappelleri [34] [35] severe ED (5 to 7 points), moderate ED (8 to 11 points), mild-to-moderate ED (12 to 16 points), mild ED (17 to 21 points), and no ED (22 to 25 points). We also used the questionnaire responses to calculate IIEF domains and assign patients to severity classes as described by Rosen [34].

### Statistical analysis

Patient and survey data were processed using IBM Corp. Released 2020. IBM SPSS Statistics for Windows, Version 27.0. Armonk, NY: IBM Corp. The statistical analysis of the means and continuous variables was per-formed using the Student’s t- test in case of normally distributed values, and the Mann–Whitney test otherwise; the differences with p–values < 0.05 were considered statistically significant, and those with p-values < 0.01 were considered highly significant. The Chi-square test was used when comparing proportions.

## Results

### Baseline characteristics

In all, 457 renal transplant patients were monitored at our transplant center during the studied period. 278 patients were excluded being patients whose graft failure required them to return to dialysis, age under 20 years or above 70 years, women, men with genital malformations incompatible with sexual activity, and the patient rejection. 179 adult male patients aged 20–70 (mean age 45.40 ± 11.5) met the inclusion criteria and were enrolled in the study. In 143 cases, the transplantation had been carried out at our center. The other 36 enrolled patients had received their grafts elsewhere but continued their long-term monitoring with us.

Fifty-four patients (30.2%) were younger than 40, while 105 were between 40 and 60 years old (58.7%), and only 20 patients (11.2%) were above the age of 60. According to the patients’ biometric data relative to well-established body mass index (BMI) thresh-olds, patients ranged from severely underweight (lowest BMI 16.98 kg/m^2^) to morbidly obese (highest BMI 37.44 kg/m^2^), and the mean BMI for the entire study group was 26.71 kg/m^2^. Socially, most of the patients (144) were in stable relationships with their partners.

Prior to the transplant, a large majority of patients (83.8%) had required dialysis: hemodialysis had been performed in 128 cases, and peritoneal dialysis in 22 cases. These histories of dialysis ranged from one month to 25 years (mean 3.36 ± 4.66 years); 53 patients had been undergoing dialysis for less than one year, and 12 patients had been dialyzed for more than 10 years due to renal impairment. The other 29 patients (16.2%) received pre-emptive transplants.

The types of donors for the patients’ renal grafts were living donors in (97 cases of whom 47.5% were relatives and 6.7% were unrelated to the patients) and cadaveric donors in 82 cases, (45.8%). Only five patients had required two kidney transplants. The mean time elapsed since the (last) transplant was 7.48 ± 5.76 years. In most cases (124), the transplant had been undertaken one to ten years prior to the study, while six patients (3,35%) had received their renal grafts more than 20 years before. By the time they were enrolled in the study, 50 patients (27.9%) had experienced one or more reversible rejection episodes and graft biopsies had been performed on 17 patients (9.5%). On enrolment, all patients had normal graft function and were receiving immunosuppressive therapy with either tacrolimus (66.48%) or ciclosporin (33.5%).

Most patients (93.5%) were undergoing treatments for various comorbidities in addition to being on immunosuppressive medication; just 11 patients (6.5%) were not taking other drugs apart from immunosuppressive drugs tacrolimus or ciclosporin. Regarding other treatments, 86.6% of the patients had hypertension and 93.3% (n = 167) required one or more antihypertensive drugs. The most used antihypertensives were calcium channel blockers (127 cases), β-blockers (110 cases), angiotensin-converting enzyme inhibitors (34 cases), and non-steroidal anti-inflammatory drugs (15 cases).

The IIEF questionnaire was completed by 170 patients, while nine patients declined to participate in this part of the study. Table 1 describes the clinical characteristics of these 170 patients, as well as transplantation and immunosuppressive treatment information.

**Table 1:** Baseline demographic, clinical characteristics, transplant data, and treatment

Characteristics	Overall (N = 170) N (%)	ED patients N (%)	Non-ED patients N (%)	Pearson chi^2^	*P* value
Age on enrolment				7.789	***0.020***
≤ 40 yrs	54 (31.8%)	23 (27.4%)	31 (36.0%)		
40–60 yrs	97 (57.1%)	46 (54.8%)	51 (59.3%)		
≥ 60 yrs	19 (11.2%)	15 (17.9%)	4 (4.7%)		
Age at transplant				11.806	***0.003***
≤ 40 yrs	101 (59.4%)	40 (47.6%)	61 (70.9%)		
40–60 yrs	65 (38.2%)	40 (47.6%)	25 (29.1%)		
≥ 60 yrs	4 (2.4%)	4 (4.8%)	0 (0.0%)		
Alcohol	17 (10.0%)	14 (16.7%)	3 (3.5%)	8.200	***0.004***
No	153 (90.0%)	70 (83.3%)	83 (96.5%)		
Smoking	32 (18.8%)	20 (23.8%)	12 (14.0%)	2.701	*0.100*
No	138 (81.2%)	64 (76.2%)	74 (86.0%)		
BMI	107 (100%)	41 (100%)	66 (100%)	1.414	*0.493*
Normal	42 (39.3%)	17 (41.5%)	25 (37.9%)		
Overweight	41 (38.3%)	13 (31.7%)	28 (42.4%)		
Obesity	24 (22.4%)	11 (26.8%)	13 (19.7%)		
Partner	114 (67.1%)	51 (60.7%)	63 (73.3%)	3.283	*0.194*
No	9 (5.3%)	6 (7.1%)	3 (3.5%)		
Not specified	47 (27.6%)	27 (32.1%)	20 (23.3%)		
Residency				0.432	*0.809*
Urban	95 (55.9%)	49 (58.3%)	46 (53.5%)		
Rural	71 (41.8%)	33 (39.3%)	38 (44.2%)		
Not specified	4 (2.4%)	2 (2.4%)	2 (2.3%)		
Type of dialysis	166 (100%)	82 (100%)	84 (100%)	7.374	***0.025***
HD	122 (73.5%)	62 (75.6%)	60 (71.4%)		
DP	17 (10.2%)	12 (14.6%)	5 (6.0%)		
Pre-emptive transplant	27 (16.3%)	8 (9.8%)	19 (22.6%)		
Time on dialysis	161 (100%)	81 (100%)	80 (100%)	6.421	***0.040***
> 10 yrs	16 (9.9%)	9 (11.1%)	7 (8.8%)		
< 10 yrs	117 (72.7%)	64 (79.0%)	53 (66.3%)		
Pre-emptive transplant	28 (17.4%)	8 (9.9%)	20 (25.0%)		
Living donor (related)	78 (45.9%)	31 (36.9%)	47 (54.7%)	7.646	***0.022***
Living donor (unrelated)	11 (6.5%)	4 (4.8%)	7 (8.1%)		
Deceased donor	81 (47.6%)	49 (58.3%)	32 (37.2%)		
Time since transplant				0.010	*0.921*
> 10 yrs	50 (29.4%)	25 (29.8%)	25 (29.1%)		
≤ 10 yrs	120 (70.6%)	59 (70.2%)	61 (70.9%)		
Family history of				0.965	*0.326*
Renal disease	27 (15.9%)	11 (13.1%)	16 (18.6%)		
No	143 (84.1%)	73 (86.9%)	70 (81.4%)		
Cause of renal failure				4.609	*0.100*
GNC	97 (57.1%)	45 (53.6%)	52 (60.5%)		
Nephropathy	28 (16.5%)	19 (22.6%)	9 (10.5%)		
Other	45 (26.5%)	20 (23.8%)	25 (29.1%)		
Hypertension	146 (85.9%)	75 (89.3%)	71 (82.6%)	1.586	*0.208*
No	24 (14.1%)	9 (10.7%)	15 (17.4%)		
Diabetes	16 (9.4%)	10 (11.9%)	6 (7.0%)	1.210	*0.271*
No	154 (90.6%)	74 (88.1%)	80 (93.0%)		
Heart disease	38 (22.4%)	22 (26.2%)	16 (18.6%)	1.409	*0.235*
No	132 (77.6%)	62 (73.8%)	70 (81.4%)		
Neurological disease	168 (100%)	83 (100%)	85 (100%)	2.865	*0.115*
Yes	6 (3.6%)	5 (6.0%)	1 (1.2%)		
No	162 (96.4%)	78 (94.0%)	84 (98.8%)		
Urological disease	31 (18.2%)	14 (16.7%)	17 (19.8%)	0.274	*0.601*
No	139 (81.8%)	70 (83.3%)	69 (80.2%)		
Organ rejection	46 (27.1%)	19 (22.6%)	27 (31.4%)	1.658	*0.198*
No	124 (72.9%)	65 (77.4%)	59 (68.6%)		
Surgical complications	14 (8.2%)	9 (10.7%)	5 (5.8%)	1.350	*0.245*
No	156 (91.8%)	75 (89.3%)	81 (94.2%)		
Chronic viral disease*				1.350	*0.245*
(Before surgery)	14 (8.2%)	9 (10.7%)	5 (5.8%)		
No	156 (91.8%)	75 (89.3%)	81 (94.2%)		
Chronic viral disease*	169 (100%)	83 (100%)	86 (100%)	1.843	*0.175*
(On transplant)	45 (26.6%)	26 (31.3%)	19 (22.1%)		
No	124 (73.4%)	57 (68.7%)	67 (77.9%)		
Comorbidities				3.729	*0.053*
(Before surgery)	105 (61.8%)	58 (69.0%)	47 (54.7%)		
No	65 (38.2%)	26 (31.0%)	39 (45.3%)		
Comorbidities (on enrolment)				1.335	*0.248*
	158 (92.9%)	80 (95.2%)	78 (90.7%)		
No	12 (7.1%)	4 (4.8%)	8 (9.3%)		
Other comorbidities	59 (34.7%)	35 (41.7%)	24 (27.9%)	3.550	*0.060*
No	111 (65.3%)	49 (58.3%)	62 (72.1%)		
Immunosuppressor therapy				0.300	*0.861*
Tacrolimus	114 (67.1%)	58 (69.0%)	56 (65.1%)		
Cyclosporine	56 (32.9%)	26 (31.0%)	30 (34.9%)		
Other medication	159 (93.5%)	78 (92.9%)	81 (94.2%)	0.124	*0.725*
No	11 (6.5%)	6 (7.1%)	5 (5.8%)		
Family history of				0.965	*0.326*
Renal disease	27 (15.9%)	11 (13.1%)	16 (18.6%)		
No	143 (84.1%)	73 (86.9%)	70 (81.4%)		

*ED* erectile dysfunction, *HD* hemodialysis, *PD* peritoneal dialysis, *BMI* body mass index, *GNC* chronic glomerulonephritis

^*^HCV-hepatitis C virus, HBV-hepatitis B virus, VPBK-polyomavirus BK, CMV-cytomegalovirus

Italic signifies *p*-value, bold italic underline indicates statistically significant *p*-value

Age, alcohol intake, type and time on dialysis, type of donor correlated positively with erectile function. Patients with ED were older than 60 compared to those with normal erectile function (p = 0.020). Even if smoking did not seem to be a significant risk factor for ED (p = 0.100), smoking was significantly associated with greater levels of ED severity (p = 0.008) and, in general, with lower scores for the domains featured in the questionnaire (intercourse satisfaction and overall satisfaction, p = 0.036). Alcohol intake was found to be a risk factor for ED, with statistical significance in both the overall IIEF score (p = 0.002) and all its domains. Obesity, the type of immunotherapy, time since the (last) renal transplant, surgical com-plications, transitory rejection episodes, causes of renal failure, presence of common risk factors (hypertension, diabetes, heart disease, neurological and urological comorbidities) did not seem to influence the presence or the severity of erectile dysfunction, as shown in Table 1.

The characteristics of erectile dysfunction reported in our study group are summarized in Table 2. Only 51 patients (30%) of patients had normal sexual function, 35 patients (20.6%) had mild ED, 57 patients (33.5%) had mild-to-moderate ED, 9 patients (5.3%9) had moderate ED, and 18 patients (10.6%) had severe erectile dysfunction. The mean total IIEF score was 16.32 ± 6.93, and the means for each domain were: 19.22 ± 7.9 for erectile function, 6.8 ± 2.9 for orgasmic function, 6.43 ± 2.1 for sexual desire, 8.96 ± 3.7 for intercourse satisfaction, and 6.78 ± 2.6 for overall satisfaction.

**Table 2:** IIEF and sexual domains general characteristic of the study group (N = 170)

	IIEF N (%)	Erectile function N (%)	Orgasmic function N (%)	Sexual desire N (%)	Intercourse satisfaction N (%)	Overall satisfaction N (%)
Severe	18 (10.6%)	14 (8.2%)	16 (9.4%)	8 (4.7%)	19 (11.2%)	13 (7.6%)
Moderate	9 (5.3%)	6 (3.5%)	10 (5.9%)	12 (7.1%)	6 (3.5%)	10 (5.9%)
Mild-to-moderate	57 (33.5%)	55 (32.4%)	50 (29.4%)	69 (40.6%)	53 (31.2%)	55 (32.4%)
Mild	35 (20.6%)	41 (24.1%)	33 (19.4%)	52 (30.6%)	66 (38.8%)	45 (26.5%)
No ED	51 (30.0%)	54 (31.8%)	61 (35.9%)	29 (17.1%)	26 (15.3%)	47 (27.6%)

### Risk factors

In Table 3, the associations between the causes of chronic kidney disease and the incidence of ED are explored. Glomerulonephritis was the most common cause of renal failure (94 cases, 55.3%), followed by hypertension (16 cases, 9.4%), focal glomerular sclerosis (16 cases, 9.4%), polycystic kidney disease (13 cases, 7.6%), congenital urological abnormalities (11 cases, 6,5%). No significant differences could be evidenced be-tween the underlying causes of the patients’ chronic renal disease and the presence of erectile dysfunction.

**Table 3:** Associations between chronic renal disease and erectile dysfunction

Causes of renal failure	Overall N = 170 (100%)	ED patients N = 84 (49.4%)	Non-ED patients N = 86 (50.6%)	Pearson chi^2^	*P* value
Glomerulonephritis	94 (55.3%)	48 (57.1%)	46 (53.5%)	0.230	*0.632*
No	76 (44.7%)	36 (42.9%)	40 (46.5%)		
Diabetes mellitus	4 (2.4%)	3 (3.6%)	1 (1.2%)	1.073	*0.365*
No	166 (97.6%)	81 (96.4%)	85 (98.8%)		
IgA nephropathy	2 (1.2%)	0 (0%)	2 (2.3%)	1.977	*0.497*
No	168 (98.8%)	84 (100%)	84 (97.7%)		
Polycystic kidney disease	13 (7.6%)	7 (8.3%)	6 (7.0%)	0.111	*0.739*
No	157 (92.4%)	77 (91.7%)	80 (93.0%)		
Hypertension	16 (9.4%)	11 (13.1%)	5 (5.8%)	2.642	*0.104*
No	154 (90.6%)	73 (86.9%)	81 (94.2%)		
Interstitial nephritis	3 (1.8%)	2 (2.4%)	1 (1.2%)	0.364	*0.618*
No	167 (98.2%)	82 (97.6%)	85 (98.8%)		
Congenital urological	11 (6.5%)	3 (3.6%)	8 (9.3%)	2.306	*0.129*
Abnormalities					
No	159 (93.5%)	81 (96.4%)	78 (90.7%)		
Focal glomerular sclerosis	16 (9.4%)	4 (4.8%)	12 (140%)	4,211	***0.040***
No	154 (90.6%)	80 (95.2%)	74 (86.0%)		
Alport’s syndrome	3 (1.8%)	2 (2.4%)	1 (1.2%)	0.364	*0.618*
No	167 (98.2%)	82 (97.6%)	85 (98.8%)		
Postinfectious	3 (1.8%)	2 (2.4%)	1 (1.2%)	0,364	*0.618*
Glomerulonephritis					
No	167 (98.2%)	82 (97.6%)	85 (98.8%)		
Lithiasis	2 (1.2%)	1 (1.2%)	1 (1.2%)	0.000	*1.000*
No	168 (98.8%)	83 (98.8%)	85 (98.8%)		
Others	3(1.8%)	1(1.2%)	2(2.3%)	0.316	*1.000*
No	167(98.2%)	83(98.8%)	84(97.7%)		

Italic signifies *p*-value, bold italic underline indicates statistically significant *p*-value

The statistical correlations between risk factors and IIEF questionnaire scores are summarized in Table 4. These results allowed us to take note of significant differences between the experience of ED for patients aged > 60 and those under 60, both in terms of total IIEF scores (12.11 ± 7.51, p = 0.025) and for each domain: erectile function (mean 14.42 ± 8.62, p = 0.020), orgasmic function (5.21 ± 2.89, p = 0.023), sexual desire (4.79 ± 2.37, p = 0.006), intercourse satisfaction (6.79 ± 4.10, p = 0.028), overall satisfaction (5.47 ± 2.65, p = 0.006). Unlike age, we could see that obesity, smoking, and alcohol intake were not significantly associated with the questionnaire scores.

**Table 4:** Correlations between risk factors and IIEF sexual domains scores (N = 170)

Risk factors (N, %)	IIEF (5–25) (mean ± SD)	EF (0–30) (mean ± SD)	OF (0–10) (mean ± SD)	SD (0–10) (mean ± SD)	IS (0–15) (mean ± SD)	OS (0–10) (mean ± SD)
Age on enrolment						
≤ 40 yrs. (54, 31.8%)	17.04 ± 6.384	20.39 ± 7.272	7.15 ± 2.750	6.59 ± 2,.261	9.22 ± 3.632	7.56 ± 2.143
40–60 yrs. (97, 57.1%)	16.74 ± 6.895	19.51 ± 7.902	6.94 ± 3.010	6.66 ± 1.925	9.24 ± 3.567	6.61 ± 2.760
≥ 60 yrs. (19, 11.2%)	12.11 ± 7.512	14.42 ± 8.624	5.21 ± 2.898	4.79 ± 2.371	6.79 ± 4.104	5.47 ± 2.653
* p*	***0.025***	***0.020***	***0.023***	***0.006***	***0.028***	***0.006***
Age at transplant						
≤ 40 yrs. (101, 59.4%)	17.52 ± 6.525	20.71 ± 7.256	7.23 ± 2.824	6.89 ± 2.068	9.57 ± 3.681	7.53 ± 2.248
40–60 yrs. (65, 38.2%)	14.92 ± 7.092	17.45 ± 8.337	6.31 ± 3.051	5.86 ± 2.053	8.23 ± 3.530	5.80 ± 2.757
≥ 60 yrs. (4, 2.4%)	8.50 ± 6.557	10.25 ± 7.974	4.50 ± 3.109	4.00 ± 2.708	5.25 ± 3.862	3.75 ± 2.630
* p*	***0.005***	***0.004***	***0.048***	***0.001***	***0.001***	***0.000***
Alcohol						
Yes (17, 10.0%)	14.76 ± 4.617	17.35 ± 5.820	6.18 ± 2.378	5.88 ± 0.993	8.65 ± 2.029	6.18 ± 1.286
No (153, 90.0%)	16.49 ± 7.134	19.42 ± 8.130	6.88 ± 3.013	6.49 ± 2.242	8.99 ± 3.853	6.85 ± 2.733
* p*	*0.078*	*0.115*	*0.179*	*0.072*	*0.166*	*0.074*
Smoking						
Yes (32, 18.8%)	15.81 ± 5.385	19.06 ± 6.565	6.63 ± 2.166	5.97 ± 1.805	8.75 ± 3.037	6.50 ± 2.185
No (138, 81.2%)	16.43 ± 7.256	19.25 ± 8.247	6.86 ± 3.117	6.54 ± 2.222	9.01 ± 3.856	6.85 ± 2.726
* p*	*0.312*	*0.583*	*0.303*	*0.102*	*0.253*	*0.183*
BMI						
Normal (42, 39,3%)	16.00 ± 7.914	19.05 ± 8.343	6.98 ± 3.250	6.71 ± 2.223	9.07 ± 4.353	6.93 ± 2.522
Overweight (28, 26.2%)	18.59 ± 6.508	21.71 ± 8.094	7.71 ± 2.883	6.63 ± 2.118	9.76 ± 3.645	7.46 ± 2.916
Obesity (45, 42.1%)	17.88 ± 5.951	21.75 ± 7.140	7.33 ± 3.046	7.17 ± 2.461	10.04 ± 2.789	7.33 ± 2.297
* p*	*0.345*	*0.252*	*0.555*	*0.454*	*0.866*	*0.401*
Family history of CKD						
Yes (27, 15.9%)	17.37 ± 7.313	20.67 ± 8.348	7.11 ± 2.847	6.33 ± 2.386	9.63 ± 3.477	7.41 ± 2.374
No (143, 84.1%)	16.12 ± 6.867	18.94 ± 7.861	6.76 ± 2.984	6.45 ± 2.119	8.83 ± 3.749	6.66 ± 2.667
* p*	*0.264*	*0.197*	*0.610*	*0.972*	*0.369*	*0.163*
Cause of CKD						
GNC (97, 57.1%)	16.66 ± 7.198	19.61 ± 7.999	7.07 ± 2.959	6.48 ± 2.185	9.03 ± 3.869	7.01 ± 2.588
Nephropathy (28, 16.5%)	14.32 ± 6.123	17.25 ± 7.085	5.79 ± 2.767	5.96 ± 1.915	8.64 ± 3.918	6.07 ± 2.448
Others (45, 26.5%)	16.82 ± 6.746	19.60 ± 8.294	6.89 ± 2.994	6.60 ± 2.240	9.00 ± 3.268	6.73 ± 2.799
* p*	*0.126*	*0.211*	***0.057***	*0.276*	*0.744*	*0.129*
Donor						
Deceased (81, 47.6%)	14.84 ± 7.100	17.85 ± 8.086	6.48 ± 2.855	5.98 ± 2.208	8.42 ± 3.731	6.21 ± 2.687
Living related (78, 45.9%)	17.77 ± 6.225	20.71 ± 7.199	7.14 ± 2.913	6.83 ± 2.035	9.62 ± 3.472	7.44 ± 2.265
Living unrelated (11,6.5%)	16.91 ± 8.723	18.73 ± 10.527	6.91 ± 3.936	6.91 ± 2.119	8.27 ± 4.692	6.36 ± 3.668
* p*	***0.042***	*0.113*	*0.251*	***0.046***	*0.062*	***0.011***
Type of dialysis						
HD (122. 73.5%)	16.17 ± 6.876	19.21 ± 7.834	6.89 ± 2.764	6.34 ± 2.111	8.91 ± 3.595	6.70 ± 2.519
DP (17, 10.2%)	14.12 ± 7.607	15.82 ± 8.719	5.29 ± 3.584	5.88 ± 2.446	7.41 ± 4.501	6.00 ± 3.000
Pre-emptive (27, 16.3%)	18.67 ± 5.818	21.63 ± 6.732	7.85 ± 2.597	7.19 ± 2.113	10.30 ± 3.099	7.85 ± 2.522
* p*	*0.076*	*0.085*	***0.027***	*0.062*	***0.045***	***0.029***
Surgical complication						
Yes (14, 8.2%)	13.71 ± 8.213	16.36 ± 9.532	6.50 ± 3.546	6.29 ± 2.555	8.57 ± 5.110	6.00 ± 3.063
No (156, 91.8%)	16.55 ± 6.788	19.47 ± 7.766	6.84 ± 2.910	6.44 ± 2.126	8.99 ± 3.577	6.85 ± 2.587
* p*	*0.162*	*0.228*	*0.843*	*0.993*	*0.736*	*0.335*
Organ rejection*						
Yes (46, 27.1%)	17.46 ± 7.070	20.65 ± 7.784	7.07 ± 3.241	6.72 ± 2.136	9.50 ± 3.704	7.11 ± 2.541
No (124, 72.9%)	15.90 ± 6.862	18.69 ± 7.963	6.72 ± 2.853	6.32 ± 2.162	8.76 ± 3.705	6.66 ± 2.662
* P*	*0.213*	*0.148*	*0.298*	*0.191*	*0.152*	*0.299*
Chronic viral disease*						
(Before surgery)						
Yes (14, 8.2%)	14.14 ± 9.181	17.14 ± 9.805	6.00 ± 3.162	6.14 ± 2.825	6.79 ± 4.823	5.86 ± 3.461
No (156, 91.8%)	16.51 ± 6.699	19.40 ± 7.762	6.88 ± 2.938	6.46 ± 2.096	9.15 ± 3.547	6.87 ± 2.540
* p*	*0.403*	*0.441*	*0.288*	*0.778*	*0.065*	*0.327*
Chronic viral disease*						
(On transplant)						
Yes (45, 26.6%)	15.13 ± 7.298	18.18 ± 8.186	6.69 ± 2.976	6.60 ± 2.136	8.76 ± 4.001	6.89 ± 2.682
No (124, 73.4%)	16.78 ± 6.790	19.65 ± 7.859	6.87 ± 2.969	6.38 ± 2.174	9.05 ± 3.622	6.76 ± 2.627
* p*	*0.139*	*0.278*	*0.730*	*0.686*	*0.769*	*0.728*
Comorbidities						
(Before surgery)						
Yes (105, 61.8%)	15.55 ± 7.010	18.40 ± 7.854	6.59 ± 2.931	6.27 ± 2.167	8.70 ± 3.935	8.70 ± 3.935
No (65, 38.2%)	17.55 ± 6.676	20.54 ± 7.961	7.17 ± 2.987	6.69 ± 2.128	9.38 ± 3.296	9.38 ± 3.296
* p*	***0.039***	***0.050***	*0.141*	*0.167*	*0.338*	*0.566*
Comorbidities (now)						
Yes (158, 92.9%)	16.09 ± 6.857	18.94 ± 7.822	6.73 ± 2.945	6.39 ± 2.12	8.87 ± 3.698	6.73 ± 2.624
No (12, 7.1%)	19.25 ± 7.569	22.92 ± 8.908	7.92 ± 3.029	6.92 ± 2.644	10.08 ± 3.825	7.5 ± 2.714
* P*	*0.073*	***0.031***	0.087	*0.221*	*0.233*	*0.189*
Hypertension						
Yes (146, 85.9%)	16.1 ± 6.856	18.93 ± 7.868	6.64 ± 2.94	6.33 ± 2.153	8.84 ± 3.703	6.71 ± 2.616
No (24, 14.1%)	17.63 ± 7.400	20.96 ± 8.322	7.83 ± 2.914	7.04 ± 2.116	9.71 ± 3.736	7.25 ± 2.723
* P*	*0.224*	*0.150*	***0.020***	*0.073*	*0.273*	*0.190*
Diabetes						
Yes (16, 9.4%)	14.63 ± 7.779	16.81 ± 9.509	6.44 ± 3.386	6.19 ± 1.797	8.13 ± 4.924	5.13 ± 3.324
No (154, 90.6%)	16.49 ± 6.843	19.47 ± 7.753	6.85 ± 2.919	6.45 ± 2.194	9.05 ± 3.569	6.95 ± 2.498
* P*	*0.377*	*0.304*	*0.677*	*0.391*	*0.844*	***0.025***
Heart disease						
Yes (38, 22.4%)	15.5 ± 7.116	17.92 ± 8.29	6.58 ± 3.244	6.29 ± 2.117	8.5 ± 3.725	6.47 ± 3.038
No (132, 77.6%)	16.55 ± 6.889	19.59 ± 7.829	6.88 ± 2.879	6.47 ± 2.174	9.09 ± 3.708	6.87 ± 2.506
* P*	*0.307*	*0.229*	*0.699*	*0.530*	*0.311*	*0.543*
Neurological disease						
Yes (6, 3.6%)	11.17 ± 7.574	13.33 ± 7.062	5.5 ± 3.271	4.83 ± 2.639	6.33 ± 3.67	633 ± 2.251
No (162, 96.4%)	16.52 ± 6.892	19.42 ± 7.922	6.85 ± 2.961	6.49 ± 2.13	9.03 ± 3.701	6.8 ± 2.649
* p*	*0.057*	***0.039***	*0.270*	*0.084*	***0.047***	*0.399*
Urological disease						
Yes (31, 18.2%)	16.9 ± 7.300	19.32 ± 8.588	7.00 ± 3.011	6.9 ± 2.241	9.13 ± 3.81	6.35 ± 2.939
No (139, 81.8%)	16.19 ± 6.869	19.19 ± 7.821	6.77 ± 2.955	6.32 ± 2.131	8.92 ± 3.699	6.88 ± 2.558
* P*	*0.486*	*0.822*	*0.573*	*0.144*	*0.671*	*0.389*
CCB						
Yes (122, 71.76%)	16.21 ± 6.461	19.18 ± 7.350	6.77 ± 2.880	6.49 ± 1.934	9.06 ± 3.515	6.74 ± 2.609
No (48, 28.24%)	16.58 ± 8.079	19.31 ± 9.361	6.92 ± 3.175	6.27 ± 2.656	8.71 ± 4.192	6.90 ± 2.707
* p*	*0.477*	*0.631*	*0.603*	*0.999*	*0.921*	*0.630*
Beta-blocker						
Yes (36, 21.18%)	15.06 ± 7.119	18.00 ± 8.312	6.42 ± 3.037	6.33 ± 1.957	8.81 ± 3.853	6.14 ± 2.958
No (134, 78.82%)	16.66 ± 6.869	19.54 ± 7.837	6.92 ± 2,.938	6.46 ± 2.213	9.00 ± 3.683	6.96 ± 2.519
* P*	0.236	0.327	0.319	0.669	0.839	0.133
Alfa-blocker						
Yes (11, 6.47)	14.27 ± 4.921	16.45 ± 5.484	6.27 ± 1.737	5.45 ± 0.688	8.27 ± 2.240	5.82 ± 1.601
No (159, 93.53%)	16.46 ± 7.040	19.41 ± 8.060	6.85 ± 3.024	6.5 ± 2.207	9.01 ± 3.790	6.85 ± 2.677
* p*	*0.091*	*0.070*	*0.189*	***0.018***	*0.119*	***0.048***
Alpha-2 adrenergic agonists						
Yes (37, 21.76%)	15.62 ± 7.013	18.57 ± 8.061	6.46 ± 2.987	6.35 ± 2.003	8.57 ± 3.819	6.54 ± 2.704
No (133, 78.24%)	16.51 ± 6.925	19.40 ± 7.928	6.91 ± 2.953	6.45 ± 2.204	9.07 ± 3.685	6.85 ± 2.616
* P*	*0.438*	*0.516*	*0.374*	*0.613*	*0.408*	*0.644*
ACE inhibitor						
Yes (31, 18.23%)	16.19 ± 8.344	19.61 ± 9.450	6.68 ± 3.400	6.39 ± 2.565	8.32 ± 4.743	6.68 ± 3.198
No (139, 81.76%)	16.35 ± 6.612	19.13 ± 7.599	6.84 ± 2.862	6.44 ± 2.065	9.10 ± 3.442	6.81 ± 2.499
*P*	*0.676*	*0.452*	*0.979*	*0.686*	*0.959*	*0.797*
Diuretics						
Yes (24, 14.11%)	15.21 ± 7.650	17.96 ± 8.579	6.00 ± 3.270	6.04 ± 1.944	8.21 ± 3.413	6.08 ± 3.063
No (146, 85.89%)	16.50 ± 6.819	19.42 ± 7.842	6.95 ± 2.893	6.49 ± 2.189	9.08 ± 3.752	6.90 ± 2.545
*P*	*0.456*	*0.460*	*0.211*	*0.309*	*0.309*	*0.254*
Antiarrhythmic						
Yes (2, 1.17%)	25.00	26.5 ± 3.536	10,00	5.5 ± 3.536	12.5 ± 0.707	10,00
No (168, 98.83%)	16.21 ± 6.908	19.13 ± 7.943	6.77 ± 2.954	6.44 ± 2.149	8.92 ± 3.710	6.74 ± 2.622
*p*	***0.024***	*0.191*	*0.074*	*0.644*	*0.094*	*0.038*
Statins						
Yes (46, 27.05%)	16.17 ± 7.103	18.80 ± 8.419	6.83 ± 2.984	6.61 ± 1.994	8.83 ± 3.732	6.04 ± 2.944
No (124, 72.25%)	16.37 ± 6.897	19.37 ± 7.785	6.81 ± 2.960	6.36 ± 2.217	9.01 ± 3.714	7.06 ± 2.460
* p*	*0.863*	*0.677*	*0.993*	*0.648*	*0.737*	***0.036***
Antidiabetic drugs						
Yes (7, 4.11%)	14.86 ± 6.492	17.86 ± 6.890	7.14 ± 2.410	7.29 ± 1.380	8.57 ± 4.077	6.29 ± 3.302
No (163, 85.89%)	16.38 ± 6.963	19.28 ± 7.996	6.80 ± 2.984	6.39 ± 2.179	8.98 ± 3.705	6.80 ± 2.608
*p*	*0.524*	*0.623*	*0.946*	*0.267*	*0.837*	*0.781*
Psychotropic drug						
Yes (3, 1.76%)	17.33 ± 5.508	19.67 ± 7.024	8.67 ± 1.528	6.67 ± 1.528	9.67 ± 2.517	6.33 ± 1.528
No (167, 98.24%)	16.30 ± 6.968	19.21 ± 7.974	6.78 ± 2.969	6.43 ± 2.169	8.95 ± 3.731	6.79 ± 2.648
*p*	0.962	0.929	0.266	0.938	0.919	0.553
NSAIDs						
Yes (15, 8.82%)	11.73 ± 6.734	13.67 ± 8.200	4.73 ± 3.081	5.20 ± 2.274	6.60 ± 3.888	5.27 ± 2.987
No (155, 91.18%)	16.76 ± 6.810	19.75 ± 7.732	7.01 ± 2.876	6.55 ± 2.114	9.19 ± 3.623	6.93 ± 2.556
*p*	***0.007***	***0.005***	***0.004***	***0.025***	***0.007***	***0.024***
Antiplatelets drugs						
Yes (7, 4.11%)	13.00 ± 9.165	15.0 ± 10.583	6.00 ± 3.512	5.43 ± 2.992	7.71 ± 4.572	5.00 ± 3.215
No (163, 95.89%)	16.46 ± 6.822	19.4 ± 7.799	6.85 ± 2.939	6.47 ± 2.115	9.01 ± 3.675	6.86 ± 2.586
*p*	*0.209*	*0.147*	*0.494*	*0.315*	*0.370*	*0.058*
Anticoagulant drugs						
Yes (6, 3.52%)	16.50 ± 4.680	18.50 ± 4.764	6.67 ± 2.422	6.33 ± 1.366	9.33 ± 1.966	6.67 ± 1.862
No (164, 96.48%)	16.31 ± 7.011	19.24 ± 8.041	6.82 ± 2.981	6.43 ± 2.182	8.95 ± 3.760	6.79 ± 2.658
*p*	*0.906*	*0.579*	*0.583*	*0.706*	*0.858*	*0.647*
Peripheral vascular disease						
Drug (pentoxifylline)						
Yes (27, 15.88%)	14.93 ± 7.631	18.07 ± 8.783	6.67 ± 3.17	5.81 ± 2.617	8.56 ± 3.955	6.63 ± 2.452
No (143, 84.12%)	16.58 ± 6.790	19.43 ± 7.785	6.84 ± 2.925	6.55 ± 2.048	9.03 ± 3.670	6.81 ± 2.669
*p*	*0.295*	*0.477*	*0.832*	*0.190*	*0.621*	*0.597*

*ED* erectile dysfunction, *IIEF* International Index for Erectile Function, *EF* erectile function, *OF* orgasmic function, *SD* sexual desire, *IS* intercourse satisfaction, *OS* overall satisfaction, *HD* hemodialysis, *PD* peritoneal dialysis, *BMI* body mass index, *GNC* chronic glomerulonephritis, *RT* renal transplant, *ACEI* angiotensin-converting enzyme inhibitor, *CCB* calcium channel blocker, *HCTZ* hydrochlorothiazide, *PPIs* proton pump inhibitors, *NSAIDs* non-steroidal anti-inflammatory drugs

^*^HCV-hepatitis C virus, HBV-hepatitis B virus, VPBK-polyomavirus BK, CMV-cytomegalovirus

Italic signifies *p*-value, bold italic underline indicates statistically significant *p*-value

The mean IIEF score of patients whose grafts had come from cadaveric donors was 14.84 ± 7.10, while for patients grafted from living donors it was substantially higher (17.77 ± 6.22, p = 0.042). The type of donor was also associated with sexual desire (mean 5.98 ± 2.20, p = 0.046) and overall satisfaction (mean 6.21 ± 2.68, p = 0.011).

The presence of comorbidities before renal transplantation appeared to interact with the overall IIEF scores (mean 15.55 ± 7.01 for patients with comorbidities vs 17.55 ± 6.68 for those without, p = 0.039). With regard to treatments, of all the categories of drugs prescribed to the patients, only the administration of NSAIDs was associated with the overall IIEF score (mean 11.73 ± 6.73 vs 16.76 ± 6.81, p = 0.007), as well as with all the domains.

At the same time, surgical complications, transitory episodes of organ rejection, family history of renal disease, instances of chronic viral disease, presence of hyper-tension, diabetes, heart disease, neurological or urological disease did not participate in statistically significant relationships with the patients’ self-assessed erectile function expressed as overall IIEF and sexual domains scores.

There are statistically significant differences between the IIEF scores collected in 2021 and 2023. The average IIEF score improved in 2023 compared to 2021 (from an average of 16.00 ± 6.881 to 17.37 ± 6.744). Statistically significant improvements were noted in the age group 40–60 years, among patients who do not consume alcohol, smoke, and had been on peritoneal dialysis for less than 10 years, with the time since transplant being also less than 10 years. Additional characteristics common to these patients include having had a transplantation procedure using an organ donated by a deceased donor, having no family history of kidney disease, the cause of renal failure being GNC, suffering from hypertension but not diabetes or other cardiovascular, neurological, or urological conditions; the transplanted organ was not rejected, there were no surgical complications or other comorbidities, but they had chronic viral diseases at the time of transplant and comorbidities before the surgery and at the time of follow-up, and they were on immunosuppressive therapy with Tacrolimus.

The IIEF score generally improved on average in 2023 compared to 2021, with some cases showing an improvement of 2 points (in individuals under 40 years old, obese, with pre-emptive transplant, without dialysis, organ donated by a deceased donor, with less than 10 years since transplant, without other urological diseases, with comorbidities before the surgery, patients who were undergoing immunosuppressive treatment with Tacrolimus or Sirolimus, by 3 points (in the three patients who received the transplant over the age of 60, in patients who consume alcohol, who had a peritoneal dialysis, with renal failure caused by nephropathy), or even more points (in single individuals, with neurological diseases, with chronic viral diseases before the surgery) as shown in Table 5.

**Table 5:** The impact of a 2-year period on the IIEF-5 score, considering different patient variables and characteristics

Characteristic	N / %	IIEF 5 score		*p*-value
		IIEF 2021 Mean ± SD	IIEF 2023 Mean ± SD	
Total lot	138 (100)	16.00 ± 6.881	17.37 ± 6.744	***0.007***
Age on enrolment				
< 40 yrs	44 (31.9)	16.80 ± 6.736	18.82 ± 5.864	*0.111*
40–60 yrs	78 (56.5)	16.03 ± 6.967	17.42 ± 6.998	***0.027***
> 60 yrs	16 (11.6)	13.69 ± 6.750	13.13 ± 6.323	*0.674*
Age at transplant				
< 40 yrs	76 (55.1)	17.09 ± 6.755	18.51 ± 6.492	*0.080*
40–60 yrs	59 (42.8)	15.03 ± 6.669	16.27 ± 6.746	*0.061*
> 60 yrs	3 (2.2)	7.33 ± 7.506	10.00 ± 6.928	*0.285*
Alcohol				
Yes	13 (9.4)	14.62 ± 4.445	17.23 ± 5.918	*0.075*
No	125 (90.6)	16.14 ± 7.083	17.38 ± 6.845	***0.022***
Smoking				
Yes	21 (15.2)	16.10 ± 4.857	18.67 ± 5.141	***0.010***
No	117 (84.8)	15.98 ± 7.200	17.14 ± 6.985	*0.057*
BMI				
Normal	28 (20.3)	16.14 ± 7.412	16.54 ± 7.406	*0.785*
Overweight	34 (24.6)	18.38 ± 6.751	18.74 ± 6.161	*0.328*
Obesity	21 (15.2)	17.48 ± 6.234	19.05 ± 6.845	*0.234*
Partner				
Yes	94 (68.1)	17.05 ± 6.403	17.09 ± 7.028	*0.540*
No	6 (4.3)	10.00 ± 9.230	18.83 ± 5.269	*0.075*
Not specified	38 (27.5)	14.34 ± 7.018	17.84 ± 6.301	***0.002***
Type of dialysis				
HD	100 (72.5)	15.94 ± 6.874	16.78 ± 6.773	*0.095*
DP	12 (8.7)	11.25 ± 6.524	14.92 ± 7.051	***0.049***
Pre-emptive transplant	24 (17.4)	18.50 ± 6.093	20.92 ± 5.340	*0.148*
Time on dialysis				
> 10 yrs	11 (8)	15.64 ± 7.487	15.91 ± 6.906	*1.000*
< 10 yrs	97 (70.3)	15.29 ± 7.003	16.67 ± 6.797	***0.019***
Pre-emptive transplant	25 (18.1)	18.68 ± 6.033	21.08 ± 5.291	*0.118*
Donor				
Living donor (related)	63 (45.7)	17.71 ± 6.241	18.38 ± 6.680	*0.331*
Living donor (unrelated)	7 (5.1)	18.00 ± 7.528	19.00 ± 9.256	*0.672*
Deceased donor	68 (49.3)	14.21 ± 7.017	16.26 ± 6.445	***0.006***
Time since transplant				
> 10 yrs	36 (26.1)	17.47 ± 6.162	16.86 ± 7.068	*0.836*
≤ 10 yrs	102 (73.9)	15.48 ± 7.072	17.55 ± 6.652	***0.003***
Family history of renal disease				
Yes	25 (18.1)	16.80 ± 7.303	18.16 ± 6.950	*0.105*
No	113 (81.9)	15.82 ± 6.805	17.19 ± 6.716	***0.021***
Cause of renal failure				
GNC	75 (54.3)	16.55 ± 6.962	17.67 ± 6.479	***0.048***
Nephropathy	25 (18.1)	13.76 ± 6.207	16.64 ± 6.745	*0.052*
Other	38 (27.5)	16.39 ± 7.016	17.26 ± 7.373	*0.432*
Hypertension				
Yes	120 (87)	15.93 ± 6.717	17.25 ± 6.723	***0.012***
No	18 (13)	16.50 ± 8.090	18.17 ± 7.023	*0.306*
Diabetes				
Yes	14 (10.1)	14.29 ± 8.287	14.79 ± 6.636	*1.000*
No	124 (89.9)	16.19 ± 6.716	17.66 ± 6.720	***0.004***
Heart disease				
Yes	32 (23.2)	14.78 ± 7.232	15.38 ± 6.671	*0.313*
No	106 (76.8)	16.37 ± 6.763	17.97 ± 6.680	***0.012***
Neurological disease				
Yes	5 (3.6)	10.80 ± 8.408	16.00 ± 8.573	*0.285*
No	132 (95.7)	16.32 ± 6.793	17.45 ± 6.715	***0.012***
Urological disease				
Yes	25 (18.1)	16.08 ± 7.702	15.88 ± 7.230	*0.782*
No	113 (81.9)	15.98 ± 6.723	17.70 ± 6.620	***0.003***
Organ rejection				
Yes	37 (26.8)	16.70 ± 7.468	18.89 ± 6.302	*0.140*
No	101 (73.2)	15.74 ± 6.673	16.81 ± 6.844	***0.031***
Surgical complications				
Yes	12 (8.7)	15.00 ± 7.781	15.83 ± 7.133	*0.582*
No	126 (91.3)	16.10 ± 6.816	17.52 ± 6.717	***0.006***
Chronic viral disease* (before surgery)				
Yes	10 (7.2)	13.60 ± 8.514	17.10 ± 6.297	***0.020***
No	128 (92.8)	16.19 ± 6.741	17.39 ± 6.800	***0.030***
Chronic viral disease* (on transplant)				
Yes	37 (26.8)	15.05 ± 6.675	16.89 ± 6.851	***0.017***
No	101 (73.2)	16.35 ± 6.955	17.54 ± 6.730	*0.089*
Comorbidities (before surgery)				
Yes	89 (64.5)	15.09 ± 7.050	17.25 ± 6.682	***0.003***
No	49 (35.5)	17.65 ± 6.300	17.59 ± 6.919	*0.680*
Comorbidities (on enrolment)				
Yes	129 (93.5)	15.83 ± 6.772	17.14 ± 6.752	***0.012***
No	9 (6.5)	18.44 ± 8.353	20.67 ± 6.021	*0.168*
Other comorbidities				
Yes	46 (33.3)	15.22 ± 6.044	16.28 ± 6.295	*0.138*
No	92 (66.7)	16.39 ± 7.263	17.91 ± 6.926	***0.023***
Immunosuppressor therapy				
Tacrolimus	92 (66.7)	15.96 ± 6.811	17.82 ± 6.605	***0.005***
Cyclosporine	45 (32.6)	16.16 ± 7.157	16.51 ± 7.076	*0.502*
Sirolimus	1 (0.7)	13.00	15.00	
Other medication				
Yes	131 (94.9)	16.18 ± 6.845	17.22 ± 6.764	***0.024***
No	7 (5.1)	12.71 ± 7.251	20.14 ± 6.122	***0.043***

*HD* hemodialysis, *PD* peritoneal dialysis, *GNC* chronic glomerulonephritis, *BMI* body mass index, *IIEF* International Index for Erectile Function

^*^HCV-hepatitis C virus, HBV-hepatitis B virus, VPBK-polyomavirus BK, CMV-cytomegalovirus

Italic signifies *p*-value, bold italic underline indicates statistically significant *p*-value, Bold italic signifies borderline *p*-value

Within this group, we identified approximately 70 patients who completed the IIEF (International Index of Erectile Function) questionnaire at both the 1-year and 2-year marks following their transplantation. The results are shown in the Table 6.

**Table 6:** The impact of a 2-year period on the IIEF-5 score

Characteristic	N / %	IIEF 5 score		p-value
		IIEF 2021 Mean ± SD	IIEF 2023 Mean ± SD	
Total lot	68 (100)	16.05 ± 6.761	17.45 ± 6.364	**0.007**
Age on enrolment				
< 40 yrs	24 (35.2)	16.76 ± 6.649	19.05 ± 5.784	0.121
40–60 yrs	38 (55.9)	15.87 ± 7.056	17.56 ± 7.006	**0.025**
> 60 yrs	6 (8.9)	12.07 ± 6.340	13.56 ± 6.656	0.592
Age at transplant				
< 40 yrs	48 (70.6)	16.99 ± 6.345	18.76 ± 6.657	0.085
40–60 yrs	18 (26.5)	14.93 ± 6.776	16.45 ± 6.634	0.069
> 60 yrs	2 (2.9)	7.56 ± 7.452	10.00 ± 6.765	0.376
Alcohol				
Yes	16 (23.5)	13.65 ± 4.334	17.45 ± 6.018	0.077
No	52 (76.5)	15.23 ± 7.156	16.45 ± 5.975	**0.021**
Smoking				
Yes	11 (16.2)	15.94 ± 5.045	18.56 ± 5.090	**0.011**
No	57 (83.8)	16.08 ± 7.345	17.45 ± 7.009	0.062
BMI				
Normal	32 (20.3)	16.98 ± 7.567	17.79 ± 7.323	0.765
Overweight	24 (24.6)	16.98 ± 6.751	17.99 ± 5.944	0.356
Obesity	12 (15.2)	15.79 ± 6.234	18.94 ± 7.097	0.222
Partner				
Yes	52 (76.5)	16.93 ± 6.345	16.97 ± 7.045	0.535
No	6 (8.8)	10.00 ± 9.023	19.02 ± 6.034	0.079
Not specified	10 (14.7)	15.02 ± 6.987	18.02 ± 5.904	**0.002**
Type of dialysis				
HD	47 (69.1)	16.03 ± 6.234	17.05 ± 6.456	0.097
DP	7 (10.3)	11.45 ± 6.168	15.03 ± 7.532	**0.047**
Pre-emptive transplant	14 (20.6)	18.34 ± 6.299	20.67 ± 5.457	0.135
Time on dialysis				
> 10 yrs	45 (66.2)	14.93 ± 7.564	14.78 ± 5.654	1.022
< 10 yrs	9 (13.2)	15.99 ± 7.785	14.22 ± 7.023	**0.022**
Pre-emptive transplant	14 (20.6)	17.52 ± 5.678	18.94 ± 6.091	0.334
Donor				
Living donor (related)	31 (45.6)	16.98 ± 6.765	18.45 ± 7.086	0.342
Living donor (unrelated)	5 (7.4)	17.96 ± 7.445	18.93 ± 9.345	0.589
Deceased donor	32 (47)	14.78 ± 6.934	16.45 ± 6.456	**0.006**
Family history of renal disease				
Yes	21 (30.9)	17.06 ± 7.786	17.96 ± 7.054	0.167
No	47 (69.1)	14.56 ± 6.546	16.95 ± 6.564	**0.022**
Cause of renal failure				
GNC	27 (39.7)	17.05 ± 7.033	17.43 ± 6.567	**0.045**
Nephropathy	22 (32.4)	14.06 ± 6.567	15.93 ± 6.853	0.057
Other	19 (27.9)	16.77 ± 6.933	16.96 ± 7.456	0.443
Hypertension				
Yes	54 (79.4)	16.03 ± 6.456	16.95 ± 6.543	**0.012**
No	14 (20.6)	15.95 ± 7.975	17.97 ± 7.876	0.375
Diabetes				
Yes	13 (19.1)	14.78 ± 8.432	15.93 ± 6.854	1.021
No	55 (80.9)	15.95 ± 6.432	17.32 ± 6.963	**0.004**
Heart disease				
Yes	12 (17.6)	15.54 ± 6.945	15.67 ± 6.345	0.333
No	56 (82.4)	16.38 ± 7.034	18.07 ± 6.765	**0.012**
Neurological disease				
Yes	4 (5.9)	11.05 ± 8.456	15.97 ± 8.789	0.277
No	64 (94.1)	15.78 ± 7.083	16.05 ± 6.323	**0.013**
Urological disease				
Yes	8 (11.8)	15.98 ± 7.564	16.08 ± 7.564	0.776
No	60 (88.2)	16.54 ± 6.342	18.06 ± 6.342	**0.003**
Organ rejection				
Yes	9 (10.3)	16.92 ± 7.675	18.93 ± 6.453	0.142
No	59 (89.7)	16.23 ± 6.897	18.06 ± 6.234	**0.031**
Surgical complications				
Yes	7 (10.3)	14.91 ± 7.543	16.03 ± 7.234	0.532
No	61 (89.7)	16.78 ± 6.765	17.23 ± 6.245	**0.006**
Chronic viral disease* (before surgery)				
Yes	5 (7.35)	14.56 ± 8.432	16.90 ± 6.567	**0.026**
No	63 (92.65)	15.98 ± 6.871	17.64 ± 6.976	**0.035**
Chronic viral disease* (on transplant)				
Yes	5 (7.35)	15.95 ± 6.567	17.09 ± 6.342	**0.019**
No	63 (92.65)	16.78 ± 7.865	18.04 ± 6.432	0.075
Comorbidities (before surgery)				
Yes	35 (51.5)	15.65 ± 7.954	16.95 ± 6.564	**0.002**
No	33 (48.5)	18.23 ± 6.234	18.29 ± 7.017	0.761
Comorbidities (on enrolment)				
Yes	62 (91.2)	16.03 ± 6.223	17.97 ± 6.451	**0.013**
No	6 (8.8)	17.31 ± 8.123	20.23 ± 6.934	0.234
Immunosuppressor therapy				
Tacrolimus	50 (73.5)	16.06 ± 6.934	18.02 ± 6.456	**0.004**
Cyclosporine	17 (25)	16.79 ± 6.023	16.78 ± 7.456	0.406
Sirolimus	1 (1.5)	14.00	13.00	
Other medication				
Yes	57 (83.9)	17.93 ± 6.267	16.62 ± 6.923	**0.042**
No	11 (16.1)	13.01 ± 7.982	19.94 ± 6.345	**0.023**

Bold underline indicates statistically significant *p*-value, Bold signifies borderline *p*-value

Table 7 presents a comparison of the severity grade of the IIEF score between the two evaluations. In 2023, there was a minor increase in the proportion of individuals without ED compared to 2021 (37.7% vs. 27.5%), as well as an increase in the proportion of patients with severe ED (13.0% vs. 10.9%). Nevertheless, there was a drop in the proportion of individuals experiencing mild to moderate erectile dysfunction (23.9% compared to 35.5%). The observed differences are not statistically significant (Pearson Chi-squared = 5.631, p = 0.228).

**Table 7:** The comparison of the two questionnaires regarding the severity level of the IIEF score

IIEF score	No ED n (%) (22–25 points)	Mild ED n (%) (17–21 points)	Mild-moderate ED n (%) (12–16 points)	Moderate ED n (%) (8–11 points)	Severe ED n (%) (5–7 points)
IIEF 2021	38 (27.5)	27 (19.6)	49 (35.5)	9 (6.5)	15 (10.9)
IIEF 2023	52 (37.7)	27 (19.6)	33 (23.9)	8 (5.8)	18 (13.0)

The majority of the patients (58.0%) remained stable in their category of erectile dysfunction, whereas 27.5% experienced an improvement in their IIEF scores, and 14.5% noticed a decline in their IIEF scores. This can be explained by the fact that although the IIEF scores demonstrated significant improvement for the majority of patients, the significance of the improvements was not substantial enough to classify them into a different evaluation category among the five categories being assessed. Therefore, the mean IIEF score for the whole group of studied patients in 2021 was 16.00 ± 6.881, indicating mild or mild to moderate dysfunction. In 2023, the value increased by one unit, reaching 17.37 ± 6.744 as showed in Table 8. This improvement is clinically significant, but it does not alter the classification of patients, as they still fall under the classification of mild erectile dysfunction. Therefore, the qualitative analysis cannot reveal statistically significant changes. This overall phenomenon is also reflected in the detailed analysis of patient subcategories selected according to the study’s proposed criteria—discrepancies are noted but are not significant enough to be deemed statistically significant.

**Table 8:** Evolution based on risk grades (improved / stable / worsened) in the 2021 IIEF questionnaire compared to the 2023 IIEF questionnaire

Characteristic	n	IIEF 5 evolution (2021–2023)			Chi-squared test
		Ameliorated n (%)	Stable n (%)	Worsened n (%)	p- value
Total lot	138	38 (27.5)	80 (58)	20 (14.5)	
Age on enrolment					*0.949*
< 40 yrs	44	12 (27.3)	27 (61.4)	5 (11.4)	
40–60 yrs	78	22 (28.2)	44 (56.4)	12 (15.4)	
> 60 yrs	16	4 (25)	9 (56.3)	3 (18.8)	
Age at transplant					*0.922*
< 40 yrs	76	21 (27.6)	45 (59.2)	10 (13.2)	
40–60 yrs	59	16 (27.1)	33 (55.9)	10 (16.9)	
> 60 yrs	3	1 (33.3)	2 (66.7)	–	
Alcohol					*0.762*
Yes	13	4 (30.8)	8 (61.5)	1 (7.7)	
No	125	34 (27.2)	72 (57.6)	19 (15.2)	
Smoking					*0.097*
Yes	21	8 (38.1)	13 (61.9)	–	
No	117	30 (25.6)	67 (57.3)	20 (17.1)	
BMI					*0.307*
Normal	28	5 (17.9)	15 (53.6)	8 (28.6)	
Overweight	34	10 (29.4)	20 (58.8)	4 (11.8)	
Obesity	21	7 (33.3)	12 (57.1)	2 (9.5)	
Partner					*0.105*
Yes	94	22 (23.4)	55 (58.5)	17 (18.1)	
No	6	4 (66.7)	2 (33.3)	–	
Not specified	38	12 (31.6)	23 (60.5)	3 (7.9)	
Type of dialysis					*0.383*
HD	100	28 (28)	54 (54)	18 (18)	
DP	12	3 (25)	9 (75)	–	
Pre-emptive transplant	24	7 (29.2)	15 (62.5)	2 (8.3)	
Time on dialysis					*0.258*
> 10 yrs	11	2 (18.2)	5 (45.5)	4 (36.4)	
< 10 yrs	97	27 (27.8)	57 (58.8)	13 (13.4)	
Pre-emptive transplant	25	7 (28)	16 (64)	2 (8)	
Donor					*0.827*
Living donor (related)	63	15 (23.8)	39 (61.9)	9 (14.3)	
Living donor (unrelated)	7	3 (42.9)	3 (42.9)	1 (14.3)	
Deceased donor	68	20 (29.4)	38 (55.9)	10 (14.7)	
Time since transplant					*0.690*
> 10 yrs	36	8 (22.2)	22 (61.1)	6 (16.7)	
≤ 10 yrs	102	30 (29.4)	58 (56.9)	14 (13.7)	
Family history of renal disease					*0.525*
Yes	25	5 (20)	17 (68)	3 (12)	
No	113	33 (29.2)	63 (55.8)	17 (15)	
Cause of renal failure					*0.169*
GNC	75	19 (25.3)	46 (61.3)	10 (13.3)	
Nephropathy	25	11 (44)	9 (36)	5 (20)	
Other	38	8 (21.1)	25 (65.8)	5 (13.2)	
Hypertension					*0.494*
Yes	120	33 (27.5)	68 (56.7)	19 (15.8)	
No	18	5 (27.8)	12 (66.7)	1 (5.6)	
Diabetes					*0.694*
Yes	14	3 (21.4)	8 (57.1)	3 (21.4)	
No	124	35 (28.2)	72 (58.1)	17 (13.7)	
Heart disease					*0.221*
Yes	32	5 (15.6)	22 (68.8)	5 (15.6)	
No	106	33 (31.1)	58 (54.7)	15 (14.2)	
Neurological disease					*0.517*
Yes	5	1 (20)	4 (80)	–	
No	132	37 (28)	75 (56.8)	20 (15.3)	
Urological disease					*0.525*
Yes	25	5 (20)	17 (68)	3 (12)	
No	113	33 (29.2)	63 (55.8)	17 (15)	
Organ rejection					*0.111*
Yes	37	9 (24.3)	26 (70.3)	2 (5.4)	
No	101	29 (28.7)	54 (53.5)	18 (17.8)	
Surgical complications					*0.414*
Yes	12	4 (33.3)	5 (41.7)	3 (25)	
No	126	34 (27)	75 (59.5)	17 (13.5)	
Chronic viral disease* (before surgery)					*0.336*
Yes	10	4 (40)	6 (60)	–	
No	128	34 (26.6)	74 (57.8)	20 (15.6)	
Chronic viral disease* (on transplant)					*0.390*
Yes	37	12 (32.4)	22 (59.5)	3 (8.1)	
No	101	26 (25.7)	58 (57.4)	17 (16.8)	
Comorbidities (before surgery)					*0.598*
Yes	89	27 (30.3)	50 (56.2)	12 (13.5)	
No	49	11 (22.4)	30 (61.2)	8 (16.3)	
Comorbidities (on enrolment)					*0.342*
Yes	129	36 (27.9)	73 (56.6)	20 (15.5)	
No	9	2 (22.2)	7 (77.8)	–	
Other comorbidities					*0.454*
Yes	46	11 (23.9)	30 (65.2)	5 (10.9)	
No	92	27 (29.3)	50 (54.3)	15 (16.3)	
Immunosuppressor therapy					*0.781*
Tacrolimus	92	28 (30.4)	51 (55.4)	13 (14.1)	
Cyclosporine	45	10 (22.2)	28 (62.2)	7 (15.6)	
Sirolimus	1	–	1 (100)	–	
Other medication					*0.428*
Yes	131	35 (26.7)	76 (58)	20 (15.3)	
No	7	3 (42.9)	4 (57.1)	–	

*ED* erectile dysfunction, *HD* hemodialysis, *PD* peritoneal dialysis, *BMI* body mass index, *GNC* chronic glomerulonephritis

^*^HCV-hepatitis C virus, HBV-hepatitis B virus, VPBK-polyomavirus BK, CMV-cytomegalovirus

Italic signifies *p*-value

The percentages of patients who experienced an improvement in their IIEF scores and changed their classification category are significantly higher than the overall rate of 27.5% for the following subcategories: smokers (38.1% improved), obese patients (33.3%), individuals who are not in a long-term relationship (66.7%), patients who received a kidney transplant from a living unrelated donor (42.9%), those whose kidney failure cause was chronic glomerulonephritis (44.0%), patients without cardiovascular diseases (31.1%), individuals who experienced surgical complications (33.3%), patients with chronic viral diseases before the intervention (40.0%) and at the time of the transplant (32.4%), and those not taking any other medications before surgery (42.9%).

Similarly, the percentages of patients experiencing a decline in their IIEF scores, resulting in a change in their classification category, are significantly higher than the overall rate of 14.5% for the following subcategories: patients aged over 60 at the time of evaluation (18.8%), individuals with normal body weight (28.6%), patients who have a stable partner (18.1%), individuals receiving HD dialysis (18.0%), patients who have been on dialysis for more than ten years (36.4%), patients whose kidney failure cause was chronic glomerulonephritis (20.0%), diabetic patients (21.4%), and individuals with surgical complications (25.0%).

The qualitative parameters identified as potential risk factors for ED (Tables 1 and 3) were incorporated into a binary logistic regression model using the Enter method to assess their interaction and establish a possible ranking. Seven risk factors were selected for analysis: age at enrollment, age at transplant, alcohol consumption, type of dialysis, duration of dialysis, donor type, and focal glomerular sclerosis. The viability of the constructed model was confirmed by the Hosmer–Lemeshow goodness-of-fit test, which yielded statistically non-significant results (p = 0.481), indicating an acceptable fit. Initially, the predictive accuracy for ED was 50.6%; however, the application of the model improved the predictive accuracy to 65.6%, suggesting that the identified predictors are valuable. Furthermore, the model accounts for 26.0% of the variance in ED, as indicated by the Nagelkerke R Square coefficient. The predictors identified are detailed in Table 9.

**Tabel 9:** Predictive factors for erectile dysfunction

Parameter	p-value	OR	95% CI for OR
Age on enrolment ≤ 40 yrs	0.593		
40–60 yrs	0.470	0.719	0.294 ÷ 1.758
≥ 60 yrs	0.778	1.299	0.210 ÷ 8.025
Age at transplant ≤ 40 yrs	0.214		
40–60 yrs	0.079	2.368	0.905 ÷ 6.196
≥ 60 yrs	0.999	9.904E + 08	
Alcohol	0.015*	7.351	1.467 ÷ 36.831
Type of dialysis HD	0.052		
DP	0.036*	4.132	1.094 ÷ 15.605
Pre-emptive transplant	0.364	0.607	0.206 ÷ 1.784
Time on dialysis > 10 yrs	0.529	0.682	0.207 ÷ 2.243
Deceased donor	0.341		
Living donor (unrelated)	0.287	0.637	0.278 ÷ 1.460
Living donor (related)	0.220	0.379	0.081 ÷ 1.784
Focal glomerular sclerosis	0.129	0.379	0.108 ÷ 1.327

The final analysis identified significant predictors, namely alcohol consumption and the type of dialysis. Specifically, patients who consumed alcohol exhibited a 7.351 increased risk of ED compared to non-consumers. Additionally, patients undergoing peritoneal dialysis (PD) demonstrated a 4.132 higher risk of ED compared to those receiving other types of dialysis.

Detailed information regarding the surgical intervention and the type of vascular anastomosis was only available for the 141 patients (78.8%) who had undergone renal transplantation at our center. In 104 of these cases (73.9%), end-to-side anastomosis was performed either with the external iliac artery or with the common iliac artery, while in the other 37 cases (26.1%) the hypogastric artery was used for the end-to-end anastomosis. The association between the unilateral interruption of hypogastric arteries and the presence or severity of erectile dysfunction was not statistically significant.

## Discussion

We performed an observational, cohort study which of 179 kidney transplant recipients monitored at a single center. Erectile dysfunction is very common in males with chronic renal disease, both in terms of prevalence (82%) and incidence (70%) [36]. In our analysis, age, alcohol use, type and duration of dialysis, type of donor, and certain classes of medicine were significantly associated with the presence of erectile dysfunction.

The observation that erectile dysfunction is highly prevalent in uremic patients is not new. Studies from the 1970s demonstrated the importance of transplantation in terms of improving quality of life and regaining potency [15, 16, 31]. Salvatierra et al. conducted a study on 130 men aged 30 to 60 who completed a sexual satisfaction questionnaire before and after kidney transplantation. The results showed significant changes in potency and libido after transplantation without significant associations with age, chronic kidney disease etiology, donor type, medications, or creatinine value [15]. Dai D Nghiem raises the issue of pelvic ischemia during surgery as a cause of impotence, but also the interruption of the hypogastric artery in cases where the arterial anastomosis of the renal graft is performed end-to-end with the internal iliac artery [31]. In our analysis of available data regarding the surgical intervention and type of vascular anastomosis (end-to-end with the internal iliac artery or end-to-side with the external iliac artery), we did not find any statistically significant associations between the presence or severity erectile dysfunction and the interruption of hypogastric arteries.

The cause of sexual dysfunction in renal transplant patients is controversial and probably multifactorial. Age, anxiety, medication side effects, interference with penile vascularity, inability to correct hormonal imbalances, or an underlying disease process are among the causative factors thought to ED [25].

As many as 90% patients with stage V chronic kidney disease experience sexual dysfunction before starting dialysis and this remains an ongoing issue for up to 60–70% of patients during the dialysis period [37]. The clinical manifestations are variable, from lack of sexual desire to the impossibility of maintaining an erection, from low levels of sex hormones to infertility, from low self-esteem to chronic depression. In a prospective study by Nassir et al., erectile function was analyzed in 52 patients before and after renal transplantation. Most kidney transplant recipients were middle aged when they had the surgery, and middle age is a time in one’s life when sexual function and fertility are still important aspects. The results of the study indicated no improvement in erectile function during dialysis but, after the kidney transplantation, patients reported a significant improvement in erectile function [13].

The prevalence rate found by Rosen for all types of end-stage renal disease patients (43% of men on hemodialysis, 25% of men on peritoneal dialysis, and 21% of men who had kidney trans-plants) was comparable to that of the general population in the U.S. for the same age categories, and higher than in Spain [4, 10, 38]. In a prospective trial, Burgos et al. observed that erectile dysfunction decreased after KT from 60 to 45%, in a prospective trial, which is different to what we found [16]. Peskircioglu et al. analyzed data from patients of a similar mean age to that of our patients (42.5 vs 45.4 in our study) and found ED in 32.3% of cases [26].

Age is a common risk factor for ED, and several studies have shown the significance regarding the frequency of ED in people who have had kidney trans-plants [39–41]. In the Mavaund trial, the average age of post-renal transplant patients with ED was 49.2 years, compared to that of patients without ED (44.5 years), similar to our patients and, in another study by El-Bahnasawy, patients with ED were substantially older (43.6 years), compared to patients without ED, whose mean age was 34.9 year. According to Wang, patients over 50 were 7.2 times more likely to have ED than younger individuals (on average, patients with ED were 55 years old and those without ED were 45.2 years old). According to Mirone, kidney transplantation can aggravate pre-existing ED in individuals younger than 45 [24].

The relationship between unhealthy habits such as drinking alcohol or smoking cigarettes and the risk of ED has been researched but not definitively elucidated. One independent risk factor for ED, according to several studies, is cigarette smoking [24]. However, the Massachusetts Male Ageing Study (MMAS) has not revealed a clear correlation between ED and smoking [10]. When comparing two groups of kidney transplant recipients with and without ED, Tian Ye found no statistically significant difference based on the patients’ smoking habits either. Regarding alcohol consumption, the MMAS research team did discover a moderately increased prevalence of ED among alcohol drinkers [10], which also emerges from our research. On the other hand, Akkaus found that alcohol use was strongly linked to a lower prevalence of ED [42]. This contrasts with our findings, which indicate that patients who consume alcohol have a greater chance of developing ED.

Regarding mental health and ED, many authors have suggested and hypothesized connection between depression and ED, suggesting that the presence of one may lead to, derive from, or worsen the other. For instance, men with ED were found 2.6 times more likely to suffer from depressed symptoms than men with normal erectile function [43, 44]. Other authors have discussed how patients who have undergone kidney transplants benefit from improved perceptions of body image after surgery, enjoying better in quality of life and sexual health [45]. Such a relationship between body image satisfaction and sexual satisfaction in CKD and transplant patients is not clear to all researchers [23]. In our study, only three patients had anxiety-depressive syndrome and were receiving anxiolytic therapy. This number is too small to allow us to contribute usefully to this debate. Also, because we lacked information from a validated questionnaire for mental conditions (de-pression, anxiety, and quality of life), we were unable to draw conclusions about the relationships between these disorders and the existence or severity of erectile dysfunction in our study.

We could not draw any conclusions regarding the relationships between mental illnesses (anxiety, depression, and quality of life) and the presence or severity of erectile dysfunction in our study due to the absence of data from a validated questionnaire including these variables. The prevalence of depression prior to and following transplantation, together with the potential correlation between mental health and erectile function, would have constituted intriguing supplementary areas of investigation had we possessed adequate pertinent data. Although our study did not include assessments of testosterone and prolactin levels, these hormones play crucial roles in sexual health, particularly in the context of aging and the presence of coexisting health conditions. Testosterone, for example, is well-known to influence libido, energy levels, and overall sexual function, while prolactin can impact sexual desire and other physiological processes. The absence of data on these hormonal profiles limits our ability to fully evaluate their contribution to the sexual health outcomes observed in our study.

In our study, on average, the patients in the ED group were older, had longer histories of dialysis prior to transplant, consumed more alcohol, had hemodialysis as a type of renal impairment, received grafts from more deceased donors, and were being treated with alpha blockers and non-steroidal anti-inflammatory drugs to a greater extent than the patients from the non-ED group. In other trials, the period of dialysis had no impact on the likelihood of ED improving, while others found that time spent on dialysis prior to transplant was an independent risk factor for sexual dysfunction [17, 41]. The vascular damage and hormonal changes brought on by dialysis therapy may explain the relationship discovered between the history of dialysis prior to transplant and ED.

Well-known ED causes include hypertension, diabetes mellitus, hyperlipemia, and uremic toxins [46]. Endothelial dysfunction may continue to manifest despite possible improvements following kidney transplantation [47]. Given that significant cardiovascular events are the primary non-infectious cause of mortality in this population of patients, these results should alert doctors to the significance of erectile dysfunction among kidney recipients since it may be a signal of cardiovascular risk [48]. In the context of established diabetes mellitus ED is hypothesized to result from the acceleration of angiopathy and neuropathy. Al Khallaf compared non-diabetic male recipients of kidney transplant to non-diabetic patients on dialysis and discovered that both groups had poor erectile function and intercourse satisfaction, but normal orgasmic function [49]. This author concluded that neither hemodialysis nor kidney transplant entirely normalize the sexual function of uremic patients. In our study, we observed that the erectile function of patients with comorbidities underwent more substantial changes. For instance, patients with hypertension had lower scores for orgasmic function, and those with diabetes had low overall satisfaction. Even though the results of associations between comorbidities and sexual function were not statistically significant, we believe that these findings are still important.

Although renal transplant may successfully cure many of the hormonal abnormalities of chronic renal failure, many male patients will still have erectile dysfunction even after a successful transplant due to the CKD-related imbalance in the hypothalamic-pituitary–gonadal axis [41, 50]. Chronic renal failure has been linked to reduced testosterone production, spermatogenesis abnormalities, and testicular impairment [16]. There is disagreement in the literature about how hormonal changes unfold following kidney transplantation. Contrary to Samsa’s findings, who reported substantial drops in mean testosterone and prolactin levels following kidney transplant, Akbari et al. discovered that hormone levels in patients receiving hemodialysis improved even normalized after renal transplantation [51, 52]. However, Tsujimura’s found that just one-third of patients experienced an improvement in general sexual performance, despite their hormone profiles returning to normal levels following transplantation [21]. Although the hormonal levels od testosterone and, prolactin were not assessed in our study, they are important for aging and concomitant conditions in the context of sexual health.

After a kidney transplant, hemodynamic alterations in the cavernous arteries have been proposed as a possible cause of ED. When the renal graft is anastomosed end-to-end to the internal iliac artery, restricting blood flow from the internal pudendal artery, this impact may be very noticeable [20]. In a retrospective analysis, Abdel- Hamid et al. found that unilateral internal iliac artery blockage had a worse impact on hemodynamic parameters in impotent recipients compared to unilateral end-to-side anastomosis to the external iliac artery [53]. However, other authors found that anastomosis performed at the internal iliac artery is in no way more preventive of ED than end-to-side anastomosis to the external iliac artery [20, 25]. According to Matheus et al., who compared two groups of kidney transplants from the perspective of arterial anastomosis, the two techniques used did not make a noteworthy subsequent difference to ED [54]. The data available in our study does not allow us to conclude on the relationship between the type of artery anastomosis and sexual function.

Because immunosuppression in transplant recipients is based on calcineurin inhibitors such as cyclosporine or tacrolimus, which are gonadotoxic drugs, other studies discuss how immunosuppressive therapy methods may negatively influence sexual function [55, 56]. Although cyclosporine is known to have a detrimental effect on endothelial function, research by Bellinghieri et al. on the influence of cyclosporine treatment on sexual hormone profiles found no association with testicular function [57, 58]. In fact, other authors found tacrolimus treatment significantly associated with normal erectile function [41]. In our study, which included patients treated with tacrolimus and cyclosporine (64.7% and 35.5%, respectively), we found no significant differences in the rate of erectile dysfunction depending on the administration of tacrolimus or cyclosporine. Instead, the patients self-reported ED scores were significantly associated with the use of alfa blockers and non-steroidal anti-inflammatory drugs.

Erectile dysfunction remains highly prevalent in kidney transplant recipients, with some studies suggesting that approximately 50% of patients experience ED after surgery. This substantial postoperative prevalence may be attributed to the surgical technique, as well as to pre-existing comorbidities (diabetes, hypertension, dyslipidemia) and unhealthy habits such as smoking, to the duration of dialysis before transplantation, to the effects of immunosuppressive or antihypertensive treatments, and to the actual underlying cause of renal failure [49, 59].

However, the effects of some of these variables have not been confirmed by our investigation; the presence and severity of ED in our patients did not correlate significantly with obesity, type of immunotherapy, time since renal transplant, surgical complications, transient rejection episodes, causes of renal failure, risk factors (hypertension, diabetes, heart disease, neurological and urological comorbidities), or we lacked sufficient data to conduct reliable analyses (anxiety, depression, hypothalamic-pituitary–gonadal axis alteration).

Alpha-blockers and NSAIDs should only be used with caution, only when absolutely required, and with full awareness of the possibility that they could impair sexual function. other drugs or treatments that might be taken into consideration.

Our study has several limitations, notwithstanding the fact that it was conducted at a single center (one of three in the country). First, patients did not complete the IIEF questionnaire at the beginning of their renal replacement treatment, but at various times post-surgery, including years later. The lack of an aetiologic description of ED is another limitation that we acknowledge. Readers should also consider the observational design of the study, the absence of hormonal profiles and other parameters with an impact on sexual function, the lack of data regarding other clinical characteristics related to the severity of the disease, as well as the lack of a control group and of a pre-transplant group. The incidence of depression both before and after transplant, as well as how mental health may have related to erectile function, would have been interesting additional areas to explore had we had sufficient relevant data.

Further study is necessary to understand why some individuals with kidney transplants experience improvement in their sexual function while others do not. Sexual response results from a complex inter-play of psychological and physiological factors like depression, anemia, diabetes mellitus, peripheral neuropathy, altered pelvic hemodynamic, and many other involving the partner, the relationship, the socio-cultural and economic context, etc. [21, 40, 60].

Further research is essential to investigate the potential advantages of treatments specifically designed to address erectile dysfunction (ED) within this population. This may include exploring the effectiveness of phosphodiesterase inhibitors (such as sildenafil), targeted counseling approaches, and lifestyle interventions aimed at improving overall sexual health. Given the notably high prevalence of ED observed even after transplantation, a more in-depth examination of these treatment options could provide valuable insights into their potential for enhancing quality of life and addressing ED-related challenges faced by these individuals. To build on the findings of this study, future research should focus on a deeper exploration of the mechanisms underlying erectile dysfunction (ED) in renal transplant recipients. Specifically, studies could aim to disentangle the roles of various contributing factors, including vascular, hormonal, and psychological influences, which may uniquely impact this population due to both their medical history and the effects of transplantation. A more granular understanding of these mechanisms may provide insight into the complex interplay between renal function, overall health, and sexual well-being.

Most transplant recipients already have several erectile dysfunction risk factors, such as diabetes and hypertension, and it is hard to assess the effects of transplantation in isolation [61]. The interaction between these factors provides meaningful context for human sexuality—re-searching this complex interplay would enable us to better understand and appreciate the role of sexual function in the overall quality of life patients with chronic conditions.

Additionally, further research is needed to assess the effectiveness of different ED treatments tailored to renal transplant recipients. This could involve evaluating pharmacological options, such as phosphodiesterase inhibitors, alongside non-pharmacological approaches like targeted psychological counseling and lifestyle interventions. Conducting comparative studies across various treatment modalities could help identify the most effective interventions for managing ED in this specific group, thereby improving quality of life and addressing a commonly overlooked aspect of post-transplant care. Future investigations with longitudinal designs, control groups, and comprehensive data collection (including pre-transplant profiles) would also enhance our understanding of the progression and treatment of ED in renal transplant recipients.

## Conclusions

Erectile dysfunction is a common sequel to chronic renal failure, but and a kidney transplant has varying effects on sexual function. The etiology is multifactorial and the two conditions share many risk factors. According to our study, the factors with significant impact on erectile function are (age, alcohol consumption, type of and time on dialysis prior to the intervention, the type of donor, and certain classes of medication (alpha blockers and non-steroidal anti-inflammatory drugs). Other factors, among which obesity, type of immunotherapy, time on current renal transplant, surgical complications, transitory rejection episodes, causes of renal failure, presence of common risk factors, as well as the usual comorbidity culprits (hypertension, diabetes, heart disease, neurological and urological conditions) did not seem to influenced the presence or the se-verity of erectile dysfunction in our patients.

Two years of follow-up revealed stable to improved erectile dysfunction, necessitating further investigation into cases where scores did not improve and the initiation of treatment in those categories of patients.

## Data Availability

No datasets were generated or analysed during the current study.
